# Peripersonal Space and Margin of Safety around the Body: Learning Visuo-Tactile Associations in a Humanoid Robot with Artificial Skin

**DOI:** 10.1371/journal.pone.0163713

**Published:** 2016-10-06

**Authors:** Alessandro Roncone, Matej Hoffmann, Ugo Pattacini, Luciano Fadiga, Giorgio Metta

**Affiliations:** 1 iCub Facility, Istituto Italiano di Tecnologia, Genova, Italy; 2 Social Robotics Lab, Computer Science Department, Yale University, New Haven, CT, United States of America; 3 Robotics, Brain, and Cognitive Sciences Department, Istituto Italiano di Tecnologia, Genova, Italy; 4 Section of Human Physiology, Ferrara University, Ferrara, Italy; Duke University, UNITED STATES

## Abstract

This paper investigates a biologically motivated model of peripersonal space through its implementation on a humanoid robot. Guided by the present understanding of the neurophysiology of the fronto-parietal system, we developed a computational model inspired by the receptive fields of polymodal neurons identified, for example, in brain areas F4 and VIP. The experiments on the iCub humanoid robot show that the peripersonal space representation i) can be learned efficiently and in real-time via a simple interaction with the robot, ii) can lead to the generation of behaviors like avoidance and reaching, and iii) can contribute to the understanding the biological principle of motor equivalence. More specifically, with respect to i) the present model contributes to hypothesizing a learning mechanisms for peripersonal space. In relation to point ii) we show how a relatively simple controller can exploit the learned receptive fields to generate either avoidance or reaching of an incoming stimulus and for iii) we show how the robot can select arbitrary body parts as the controlled *end-point* of an avoidance or reaching movement.

## Introduction

The peripersonal space (PPS) is of special relevance for the life of any complex animal. When objects enter the peripersonal space, they can be reached for, grasped, or be a threat, evoking for example an avoidance response. Peripersonal space thus deserves special attention and probably justifies the specific neural circuitry devoted to its representation. The brain has to dynamically integrate information coming from several modalities: motoric, visual, auditory or somatosensory. In primates, the evidence derived primarily from recordings in the macaque identifies a specific fronto-parietal network of neurons as the circuitry responsible for the representation of peripersonal space, as well as the connection to extant behavior (e.g., [1–3]). In the frontal lobe, the principal convergence locus has been discovered to be area F4 of the ventral premotor cortex [4–6] including the region of the spur of the arcuate sulcus [7]. In the parietal lobe, the area most strongly connected to area F4 is area VIP (Ventral Intraparietal [8]). In spite of the fact that observations report the presence of auditory responses [9], in this work we leave audition aside and focus instead on the integration of visual and tactile inputs.

A large part of peripersonal space coding can presumably be attributed to populations of polymodal neurons that, in addition to motor discharge, have tactile and visual receptive fields (RFs). Visual RFs usually extend from the tactile ones in the space around the respective body segment (see e.g. [4, 5]; for a review, see [1–3]). Furthermore, the visual RFs are often coded in the same frame of reference (FoR) of the corresponding body part and, therefore, during active or passive mobilization, they move with the body part in 3D space. This suggests that motor and proprioceptive information is integrated in a body-part-centered encoding. A good part of the evidence coming from the monkey is presumably informative in the case of humans as well [10].

Timely and appropriate object-directed actions in the peripersonal space are crucial for the survival of the animal. Depending on the context, actions may constitute either an approaching or an avoidance behavior. In the case of avoidance behavior, this creates a “margin of safety” around the body, such as the flight zone of grazing animals or the multimodal attentional space that surrounds the skin in humans [2]. An analogous behavior is desirable in general-purpose robots as well, when significant interaction is expected to happen in unconstrained environments. However, to date, robot controllers largely concentrate on the *end-point* as the only part that enters in physical contact with the environment. The rest of the body is typically represented as a kinematic chain, the volume and surface of the body itself rarely taken into account. Sensing is dominated by “distal” sensors, like cameras, whereas the body surface is “numb”. As a consequence, reaching in cluttered, unstructured environments poses severe problems, as the robot is largely unaware of the full occupancy of its body, limiting the safety of the robot and the surrounding environment. This is one of the bottlenecks that prevent robots from working alongside human partners.

While individual components that presumably constitute the representations of space around the body can be studied in isolation using computational models in simplified (for example 2-dimensional) scenarios, their interactions are difficult to model without an articulated body with corresponding sensorimotor capacities and actual interaction with the environment. Indeed, in animals and humans, these representations are gradually formed through physical interaction with the environment and in a complex interplay of body growth and neural maturation processes. Self-touch (also called double-touch) is presumably one of the behaviors that impact the formation of multimodal body representations. For example, “*by 2-3 months, infants engage in exploration of their own body as it moves and acts in the environment. They babble and touch their own body, attracted and actively involved in investigating the rich intermodal redundancies, temporal contingencies, and spatial congruence of self-perception*” [11]. Such behaviors may initially be reflexive and controlled by spinal circuitry—the wiping/scratch reflex has been demonstrated in frogs [12, 13], though its existence is debated in humans [14]—but progressively become more complex and voluntary. These contingencies and congruences that arise occur across different motor and sensory modalities, with the motor/proprioceptive and tactile starting already in prenatal stage. Vision is presumably incorporated later, during the first months after birth hand in hand with the maturation of the visual system (see e.g., [15]). Perhaps even later, contingencies will encompass external objects (this loosely resembles the sensorimotor stage of development put forth by Piaget, e.g. [16]).

This simplified developmental timeline constitutes the skeleton of our work in the humanoid robot. The humanoid in question is the iCub, a child robot designed to support studies on artificial cognitive systems [25]. The iCub has a human-like morphology and a subset of the sensory capacities of the human body. Lately, it has been equipped with a set of tactile sensors [26], which provide information about local pressure upon contact with an object or, generically, any part of the environment. We are concretely in the position of studying how motor-proprioceptive-tactile and visuo-tactile associations are developed via an artificial learning process. The robot can and will therefore establish a margin of safety by interacting with its own body and the environment, extending its cutaneous tactile surface into the 3D space surrounding it. An overview of the developmental timeline is provided in Tables 1–4, with putative developmental milestones in the left column and their robotic counterparts on the right.

**Table 1:** Developmental milestone 1: *“Bare” or “blind” double-touch*.

	Developmental Milestone	Robotics Implementation
**1a.**	**Double-touch from body babbling or mediated by reflexes**. Fetuses as well as infants spontaneously contact their bodies, giving rise to self-touch events. Correspondence between *motor* (how to command a limb to touch a specific body part) and *tactile* (cutaneous stimulation on touching and touched body part) information are established [11, 17].	**Double-touch using inverse kinematics**. In the iCub robot, we used a solution for double-touch developed in Roncone et al. [18]. This capitalizes on an existing kinematic model of the robot as well as calibration of the artificial skin with respect to a common FoR and employs a modified inverse kinematics solver. This solution automatically encompasses different arm configurations, since current joint positions automatically enter the kinematic representation. *Subject to learning*: As one arm approaches and eventually contacts another body part (the contralateral arm in our case), the position and velocity of the approaching arm are acquired from current joint angle values and kinematic model of the robot and then remapped into the FoR of the taxels on the touched limb. These taxels then, in parallel and in their individual FoRs, learn a probabilistic representation of the likelihood of a stimulus—the approaching limb in this case—contacting them.
**1b.**	**Invariance with respect to the configuration of the “touched” limb**. If movement is directed to a body part that can assume different configurations with respect to the body frame (e.g. arm), the information about the current position of this body part needs to be taken into account—presumably using *proprioceptive information*. Some form of remapping of tactile information into external (to the skin) reference frames seems necessary (see Heed et al. [19] for a review). *Outcome*: Prediction of double-touch from motor/proprioceptive information.	

**Table 2:** Developmental milestone 2: *Double-touch with vision*.

	Developmental Milestone	Robotics Implementation
**2a.**	**The motor/proprioceptive information about the position of the approaching arm is augmented by vision**. Here we assume that its 3D position with respect to a certain reference frame can be retrieved (using stereopsis). This can then be used to develop *visuo-tactile associations* able to predict incoming contact based on visual information.	**Visual tracking with extraction of 3D coordinates; head and eye kinematics**. A visual tracker (specified in the following sections) is used to detect and extract coordinates of approaching limb into a body-centered reference frame. A model of eye and head kinematics together with current joint values are used to perform the necessary kinematic transformations. Further, a gaze controller [20] is employed to track the approaching stimulus (fingertip in this case). Different limb as well as head and eye configurations are automatically taken into account. *Subject to learning*: Probabilistic representation of stimuli eventually resulting in double-touch, but this time utilizing visual information about the approaching limb.
**2b.**	**Invariance with respect to the configuration of the “touched” limb**. Similarly to **1b**, if the “touched” body part can assume different configurations, this needs to be taken into account in order to register the visuo-tactile association correctly—presumably in a reference frame centered on the body part. Again, *proprioceptive* signals about limb configuration can provide this information.	
**2c.**	**Invariance with respect to the head and eye configuration**. The touching limb needs to be followed in space by gaze. For correct registration of the active limb’s position and subsequent coordinate transformations, *proprioceptive* information about the current neck and eyes configurations is needed (see [21, 22] regarding the role of gaze in reaching to somatosensory targets). *Outcome*: Prediction of double-touch while extracting the position and velocity of the approaching arm from visual information.	

**Table 3:** Developmental milestone 3: *Visuo-tactile associations pertaining to external objects*.

	Developmental Milestone	Robotics Implementation
**3a.**	**Tactile-visual-proprioceptive learning from any approaching stimulus**. The tactile-visual-proprioceptive association learned in previous stage is generalized and applied to any objects nearing the skin. Visual perception of own approaching body parts is substituted by detection and tracking of moving objects in the body surroundings. We did not consider further stimuli approaching the face (e.g., [23]), where expanding optic flow fields may in fact be at use (e.g., [24]), but objects nearing the limbs. *Outcome*: Prediction of contact of skin parts—with own body or with generic objects—using visual information.	Same as Milestone **2** above, but using a different visual perception pipeline able to accommodate arbitrary objects (detailed in the paper). *Subject to learning*: Probabilistic representation of stimuli eventually resulting in contact with the skin, utilizing visual information about approaching objects.

**Table 4:** Developmental milestone 4: *Exploitation of learned associations*.

	Developmental Milestone	Robotics Implementation
**4a.**	**Avoidance behaviors**. Prediction of contact is exploited to trigger coordinated avoidance behaviors w.r.t. either the own body (i.e. avoiding self-collisions) or the external world (i.e. avoiding incoming potentially harmful objects) (e.g., [2]). *Outcome*: Effective “safety margin around the body”.	**Distributed avoidance / catching controller**. Taxels with activation above a certain threshold contribute to a resulting movement vector that is executed by a Cartesian controller. The avoidance / reaching differs only in the direction of the final movement vector.
**4b.**	**“Reaching” with arbitrary body parts behaviors**. The peripersonal space representation facilitates actions toward nearby objects, allowing to reach for them with any body part. *Outcome*: reaching actions with arbitrary body parts.	

The robotics implementation departs in many respects from the mechanisms that presumably operate in the primate brain. The correspondence between biology and robotics is often established at a behavioral level rather than in the details of the implementation. In particular, for mostly practical reasons, we assume that the robot’s kinematics and mapping of tactile information into reference frames is given. The implementation of the double-touch behavior itself (from [18]) is taken as a primitive. Conversely, learning/calibration of the spatial receptive fields around individual taxels (tactile elements) is primarily addressed here and relates to biology.

Building on the developmental pathway outlined above, we model peripersonal space on a humanoid robot equipped with full-body tactile sensors. Our model keeps in register each “spatial” visual RF to a taxel of the robot’s skin. Starting from an initial “blank slate”, the distance and velocity of a stimulus entering any given RF is recorded, together with information on whether the object had eventually contacted the selected tactile element. Distance and velocity of the stimulus are measured with respect to each taxel, in real time and in parallel. In this model, RFs are proxies for the neural responses, each of them represented by a probability density function. Probabilities are updated incrementally and carry information about the likelihood of a particular stimulus (e.g. an object approaching the body) eventually contacting the specific taxel at hand. We use the distance to the taxel and its time to contact (distance/velocity) to compactly identify the stimulus in a bi-dimensional parameter space.

For learning probabilities, we explore two stimulation modalities: i) self-touch and, more generically, ii) objects moving toward the body surface. In the first case, the stimulus is generated autonomously by the robot—for example, a finger touching the contralateral arm. The robot executes self-touching behaviors and uses proprioceptive signals to measure the approach kinematics, which in turn constitute the training set to estimate probability densities. In the second modality, stimuli are generated by any object in the vicinity of the body surface and perceived visually and through its contact with the skin.

There are a number of computational models addressing phenomena related to peripersonal space representations. A major component of many of them is coordinate transformations, which seem inevitable in order to code visual information in body-part centered FoRs; this has been investigated extensively and several connectionist models have been proposed (e.g., [23, 27, 28]). On the other hand, Magosso et al. [29] took FoR transformations for granted and focused on the mechanisms of tactile and visual interaction. They proposed a neural network that models unimodal (visual and tactile) and bimodal representations of an imaginary left and right body part and demonstrated a number of phenomena reported in humans (e.g. tactile extinction). Some of the studies targeting body schema and peripersonal space representation models were reviewed in Hoffmann et al. [30]. Since platforms with tactile sensing are rare, most of the work has focused on the interaction of visual and proprioceptive information (in robotics typically equated with joint angles from encoders). For example, Antonelli et al. [31, 32] developed models in different humanoid robots, focusing mainly on peripersonal space in the sense of space within reach and the visual aspects thereof. A number of embodied models were also developed by Asada and colleagues. Hikita et al. [33] used a humanoid robot and employed a bio-inspired architecture (self-organizing maps, Hebbian learning, and attention module) to learn the visual receptive field around the robot’s hand and its extension when using a tool—inspired by the behavior of the “distal” type neurons reported by Iriki et al. [34]. Touch was only emulated and used to trigger the visuo-proprioceptive association. Finally, most related to our approach, Fuke et al. [35] used a simulated robot touching itself on the face to model the putative mechanism leading to the visual and tactile response properties of neurons in the ventral intraparietal area (VIP). A hierarchical architecture with visual, proprioceptive and tactile modality was used. After learning, as the robot’s hand approached its face, contact with the skin could be predicted.

In robotics, safe interaction, especially when involving humans, is a crucial need of future assistive machines. There is necessity for technologies that allow robots to acquire some form of “whole-body” and “nearby-space” awareness. Traditionally, a significant body of work has been produced in the context of obstacle-avoidance planners, able to compute safe end-effector trajectories off-line if provided with complete knowledge of a static environment and a precise kinematic model. These approaches fall short in presence of modeling errors or when environments change dynamically. To this end, the classic planning techniques had to be complemented by reactive strategies such as the potential field approach [36]. More recently, frameworks taking the whole occupancy of a robot body into account have appeared: Flacco et al. [37] proposed a motion controller with online collision avoidance for both end-effector and the manipulator body; Frank et al. [38] proposed a modular framework (MoBeE) where a planner can be overridden by a reactive controller. Still, the performance of systems relying on distal sensing (such as from cameras or depth sensors) degrades if the perception of the environment is not reliable or the model of the robot kinematics inaccurate. A feedback loop that is as close as possible to the interaction itself is needed.

In recent years, tactile systems have been proposed as a way to close the loop precisely where the interaction occurs. However, the lack of suitable platforms limits research in this direction: although diverse tactile sensing technologies have been developed (see [39] for a review), robots with whole-body tactile sensing have been mostly unavailable. Alternative solutions relied on force/torque sensing and impedance control schemes that ensure compliant behavior of the platform on contact (e.g., [40]). Shimizu et al. [41] used force/torque feedback together with encoder information to develop self-protective reflexes and global reactions for the iCub robot. Distributed sensing over the whole surface of a robotic manipulator was used by Mittendorfer and Cheng [42]. Utilizing information from accelerometers from their multimodal “skin” during a motor exploration phase, the direction of movement of every sensory unit in response to every motor could be learned. Activations of infra-red distance sensors on the same sensory unit could then be used to trigger local avoidance reflexes to approaching objects. Finally, Jain et al. [43] devised a controller that allows for reaching in clutter while taking into account multiple contacts and keeping the forces within set limits. The solution was verified on a robot featuring a tactile-sensitive forearm. However, solutions combining interaction-based and contact-less (distal sensing) approaches are rare ([44] being a notable exception). This is where our work exploiting visual and whole-body tactile information ties in.

In this work, we set forth to implement a model of peripersonal space that includes self-tuning abilities in the form of learning from examples. Specifically, we do not model the acquisition of the FoR transformations but rather we focus only on the construction of the responses of the RFs. We build on our previous work [45], where we presented a simplified version of the model dealing solely with approaching external objects and registering their distance. Here we extend this work by presenting a complete developmental timeline, in which examples are first collected through self-exploration or self-touch, resulting in concurrent motor-tactile and visuo-tactile stimulation of different areas of the body. This is then complemented by external approaching objects. Furthermore, the RFs’ representations take into account the time to contact of the incoming stimulus. Finally, the acquired RFs are used in a controller to implement avoidance and reaching behaviors thus implicitly testing their performance.

This article is structured as follows. In the Results section, the properties of the proposed model are first verified in simulation (Section Learning in a single taxel model) and then on the iCub (Section Learning in the real robot). Finally, the peripersonal space representation is used to generate avoidance as well as “reaching” behavior using arbitrary body parts of the robot (Section Exploitation of the learned associations). This is followed by Discussion and Conclusion, which contains a summary, limitations of the model and future work. A detailed description of the experimental setup and the proposed computational model is presented in Section Materials and Methods.

## Results

Results from four different experimental scenarios are reported (we refer the reader to Tables 1–4 above for an overview of the developmental timeline). First, the behavior of the proposed representation is studied in a simulated single taxel model (Section Learning in a single taxel model). Second, we demonstrate how the robot can learn tactile-motor and tactile-visual representations via a double-touch scenario and by tracking arbitrary objects as they near the skin. Finally, the utility of the learned representations is demonstrated in the avoidance and reaching scenario that exploits the tactile-visual representations learned previously. The source code has been released online with an open source license and it is readily available for any iCub robot [46]. All the relevant data and scripts needed to reproduce the results shown are accessible at the public repository [47].

### Representation of “Space Around the Body”

**Fig 1:**
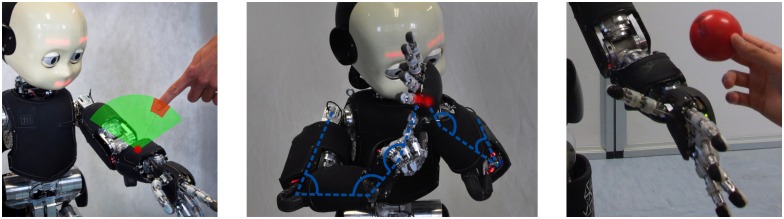
Illustration of the setup of different scenarios. **(left)** Receptive field above one of the left forearm taxels. **(middle)** iCub double-touch behavior with a simplified schematic of the kinematics and joint angles. **(right)** An object approaching the left forearm.

We have chosen a distributed representation whereby each taxel learns a collection of probabilities regarding the likelihood of being touched by a moving object. The physiology of the observed neural RFs suggests that their extension in space is modulated by the speed of the incoming stimulus. In addition, the relative position of the stimulus with respect to the receptive field (RF) clearly determines the activation strength of a given neuron. Inspired by these considerations we define a parameter space of two variables: (i) distance from the taxel *D*; (ii) time to contact *TTC*. *TTC* is calculated from the distance *D* and velocity of the incoming stimulus. Fig 1 illustrates the receptive field around one taxel of the forearm and the two main scenario types: self-touch and an external object approaching toward the body. *D* and *TTC* can be calculated in the reference frame of each taxel. Practically, this is possible because of the existing calibration procedure of the robot skin due to del Prete et al. [48] and a full model of the robot’s kinematics derived from CAD data, including the head and eyes [49]—as detailed in the Experimental Setup Section. For stimuli perceived visually, additional processing involving stereo vision is required. In fact, any observation is mapped into the iCub Root FoR (located around its waist) and subsequently transformed to the reference frames of individual taxels. It is important to note that measurements are affected by parametric errors in addition to their intrinsic measurement noise (the modeling errors are discussed in detail in Section Kinematic model and coordinate transformations). The effect of modeling errors can be, for example, that stimuli that are in physical contact with the skin can be perceived as seemingly penetrating the robot surface when employing a sequence of coordinate transformations using the kinematic model and current joint angle measurements. Subsequently, this results in a negative measure of distance *D* with respect to the taxel surface normal. Conversely, if the errors bring about an offset in the opposite direction, an actual contact on the robot’s skin may correspond to a perceived positive distance. Our training data will be affected systematically by these errors which reflect onh the estimated probability densities.

### Learning in a single taxel model

**Fig 2:**
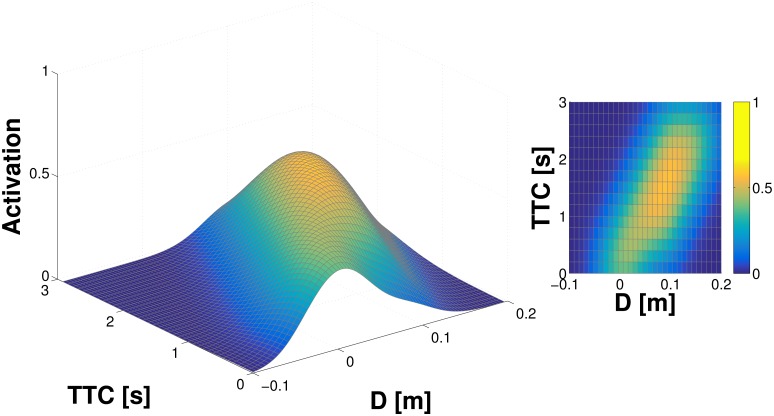
Representation learned in single taxel model. *D* and *TTC* estimated from distance and velocity of the object. **(Left)** Full 3D graph of the representation. The *z*−axis is given by the activation—estimate of the probability of object eventually landing on the taxel. **(Right)** 2D projection; third dimension preserved in the color map.

The properties of the learning procedure as well as the proposed representation are investigated in a single taxel model (as specified in Section Monte Carlo simulation of a single taxel). The results from 500 iterations of the simulation—500 objects being “thrown” toward the taxel—are illustrated in Fig 2. They show the representation of the “probability density” (it is not a real probability density—see Section Internal representation) after learning and smoothing using the adapted Parzen window method: the full landscape on the left and its projection in 2D with color coding (the probability of contact) on the right. A clear “ridge” can be seen in both plots, which corresponds to the trajectories of objects as they approach the taxel and both *D* and *TTC* are decreasing. The contact with the taxel occurs at both *D* and *TTC* equal to 0.

**Fig 3:**
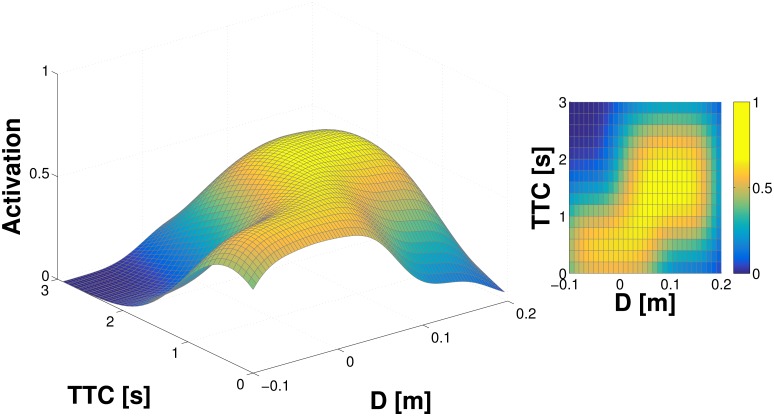
Representation learned in single taxel model with noise and systematic error (−10*cm* offset). See text for details.

In a second simulation, in order to better approximate the experimental conditions encountered by the real robot, two additional features are added to the model. First, Gaussian noise is added to the measurement of position and velocity (and hence *D* and *TTC*). Second, we account for the fact that the object position and velocity measurements in the real robot are subject not only to random, but also to systematic errors. In particular, in both tactile-motor (double-touch) and tactile-visual scenarios, the coordinate transformations needed to map the approaching object to the FoR of individual taxels rely on the model of the robot kinematic structure and its visual system, which are subject to errors (see Section Kinematic model and coordinate transformations). To clearly demonstrate the effect of this on the representation, we introduce a significant systematic offset (−10*cm*) to the simulation. The results for this configuration—noise and systematic error—can be seen in Fig 3. The Gaussian noise results in an overall broader profile of the activation landscape. The offset can be clearly seen in the distance axis, with the “ridge” of high activations cutting the *x*−axis in the negative domain.

### Learning in the real robot

The proposed method is then tested in a real-world setup where real, physical stimuli approach the iCub’s skin. We investigate the learning of peripersonal space representations in three scenarios corresponding to the putative developmental milestones as discussed in the Introduction. Initially, learning involves exclusively tactile and motor signals (cf. Table 1) as induced by self-touching behaviors (Section Tactile-motor learning: double-touch). In the second phase—Section Tactile-visual learning from double-touch, visual information replaces motor information about the “touching” arm (corresponding to Table 2). Finally, this approach is generalized to any incoming external stimulus that contacts the skin (Table 3) in Section Tactile-visual learning using external objects. An overall comparison of the representations learned in the different scenarios as well as an analysis of the learning process for two adjacent taxels is shown in Section Interim discussion on learning in the real robot.

Table 5 provides a quantitative overview of the data sets collected in the three scenarios. In every case, the skin parts involved are listed, along with the experimental time elapsed, number of trials (i.e. the number of independent stimuli nearing the robot’s skin), and total number of samples (an average of 37 samples per trial were recorded). The movements were directed toward the internal part of the left forearm in all the scenarios—this skin region facilitates the self-touch behavior in the robot—with the same 8 taxels subject to learning. In addition, to demonstrate the generality of the approach, the outer part of the left forearm (4 taxels) as well as the right hand (skin on the palm, 4 taxels) were targeted in the tactile-visual scenario with external objects.

**Table 5:** Learning in the real robot. Comparison between three experimental sessions performed on the iCub robot. For each session and each body part under consideration, the elapsed time in minutes (**ET**), the number of trials (**#T**), and the total number of input samples (**#S**) are shown. See text for details.

Experiment	Body Part								
	Left Forearm (internal)			Left Forearm (external)			Right Hand		
	ET[min]	#T	#S	ET[min]	#T	#S	ET[min]	#T	#S
Tactile-motor	31	82	3512	–	–	–	–	–	–
Tactile-visual (double-touch)	30	45	1166	–	–	–	–	–	–
Tactile-visual (ext. objects)	23	53	1886	17	34	1348	44	77	2833

It is worth noting that the data collection and learning process was fast (summing up to 142 minutes for all the experiments reported together). In fact, even a single positive (i.e. touch of the skin) trial gives rise to a usable representation (cf. Section Interim discussion on learning in the real robot below). This is considered a significant merit of the proposed approach, since the algorithm can be used on-line and in real-time without an *a priori* batch learning session: the peripersonal space representations immediately provide prior-to-contact activations and are then refined over time. The smoothing approach used (Parzen window applied to the discrete domain) is specifically responsible for this in the context of undersampled spaces.

### Tactile-motor learning: double-touch

**Fig 4:**
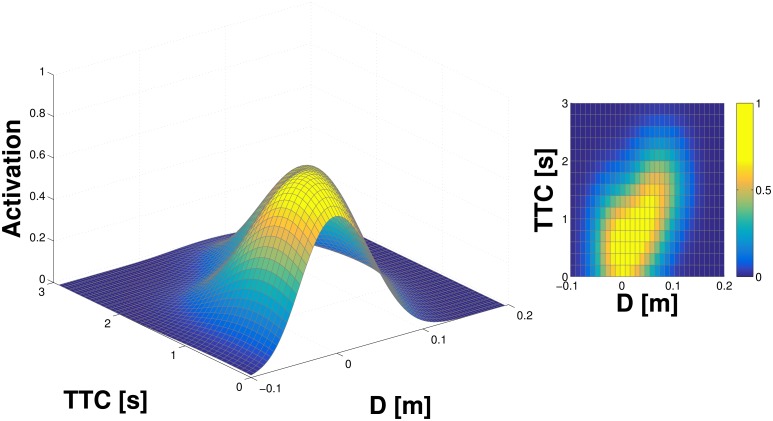
Tactile-motor representation learned in the double-touch scenario. Results for taxel nr. 2 on the inner part of the left forearm. See text for details.

The first experiment on the real robot deals with the developmental milestone described in Table 1—“bare” or “blind” double-touch. In this experiment, we used the controller developed in Roncone et al. [18]. The robot is stimulated by touching it on the forearm; see Fig 1 (middle) for a schematic illustration. A modified inverse kinematics solver and controller finds a solution whereby the contralateral fingertip touches the stimulated taxel, and commands both arms to the respective pose (note that the taxel eventually touched by the robot may differ from the one that was initially stimulated because of the systematic errors). Importantly, the robot configuration may differ at each trial, depending on the inverse kinematics solution found by the solver. After the double-touch event, a buffer is used for data collection and learning as explained in Section Data collection for learning. That is, the kinematic model and the joints configuration at every time step are used to convert the position of the tip of the index finger (the approaching body part) to the FoRs of the taxels on the approached and eventually touched part. Unfortunately, only a subset of the skin is physically reachable by the robot—some configurations are kinematically not feasible or unsafe. Therefore, for our experiments, we selected eight taxels (as explained in Section Artificial skin) on the inner part of the forearm for which the double-touch behavior was triggered. These eight taxels updated their representations in parallel using the distance and expected time to contact as the contralateral finger was approaching. As detailed in Table 5, there were 82 successful double-touch trials, with a total of 3512 training samples. That is, there were 82 trajectories sampled at *T* = 50*ms* that resulted in a contact with the selected area of the skin. From the eight taxels considered, only six were actually touched at least once by the contralateral index finger. In all of them, the results after learning were qualitatively similar and matched the predictions of our model. The results for one of the taxels with the largest number of training samples (taxel nr. 2; 1625 samples) are shown in Fig 4 and, in fact, they demonstrate learning of a tactile-motor margin of safety: i.e. prediction of self-collisions in the absence of visual input. No offset in the position is reflected in the learned representation, indicating that the model of the kinematic loop connecting the two arms was reasonably accurate.

### Tactile-visual learning

With respect to visual learning, two experiments were performed: (i) the double-touch scenario was repeated, but in this case, utilizing visual input rather than the “motor” information of the moving arm (corresponding to the milestone in Table 2); and (ii) independently moving objects nearing the robot’s body were used (Table 3). In both cases, the stimulus (robot fingertip or the moving object) was detected, tracked and its trajectory prior to contact recorded. The position and velocity of the stimulus was extracted and remapped first into the iCub Root FoR and eventually into the FoR of individual taxels, yielding the [*D*, *TTC*] pairs used for learning the representation of nearby space in the corresponding taxels.

#### Tactile-visual learning from double-touch

**Fig 5:**
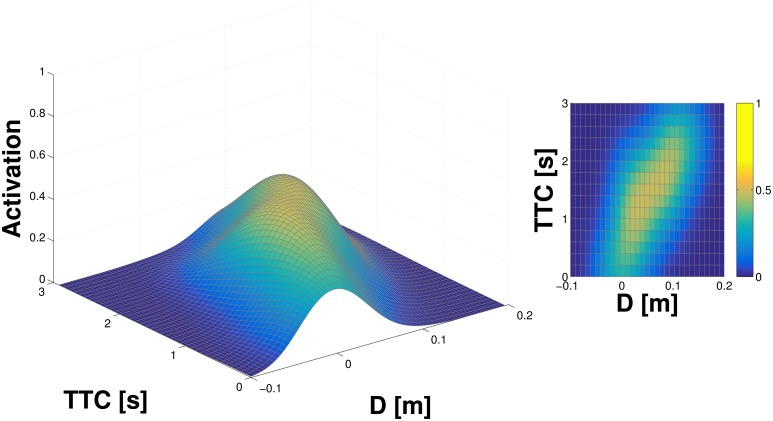
Tactile-visual representation learned in double-touch scenario. Results for taxel nr. 2 on the inner part of the left forearm. See text for details.

For this variant of the scenario—double-touch with the moving finger perceived visually—we added a small colored marker to the fingertip that was commanded to execute the double-touch movement. The method to extract the finger’s coordinates is described in Section Visual processing and gaze control—“Tracking of fingertip with colored marker”. The learning procedure was exactly the same as in the double-touch scenario described earlier. We performed 45 trials. The results show a similar pattern to the previous case; the same taxel (nr. 2; 376 samples) on the inner forearm is selected for illustration in Fig 5.

#### Tactile-visual learning using external objects

**Fig 6:**
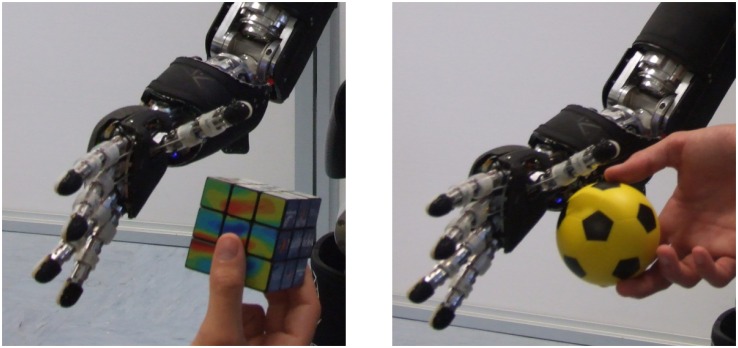
Objects approaching right palm. **(Left)** Cube. **(Right)** Small ball.

This case is a generalization of the double-touch experiments whereby the stimuli are generated by visually perceiving an approaching object that eventually touches the body surface. In this session, tactile-visual trials are carried out by a human experimenter that manually approaches the robot’s skin with a series of objects. The visual processing pipeline is explained in Section Visual processing and gaze control—“Tracking of generic objects”. This setup was validated using two objects, a cube and a small ball (see Fig 6), approaching the taxels on the robot’s body. Importantly, we were no longer limited to parts of the skin that can be activated in the self-touch configurations. We have extended learning to the outer part of the left forearm as well as palm of the right hand.

**Fig 7:**
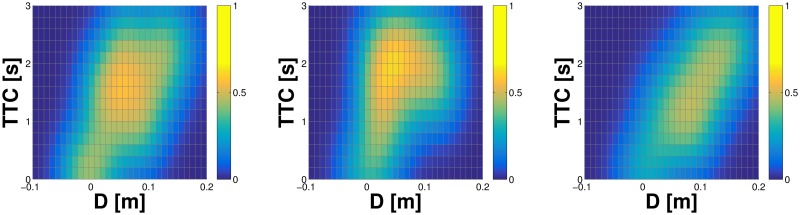
Tactile-visual representation learned from oncoming objects. **(Left)** Inner part of left forearm (taxel nr. 2). **(Middle)** Outer part of left forearm (taxel nr. 8). **(Right)** Right hand (taxel nr. 2). See text for details.

On the inner part of the left forearm, the same eight taxels of the previous scenarios were considered. Additionally, four taxels on the outer part of the forearm and four taxels of the right palm were also stimulated. We conducted a total of 53 trials for the inner part of the left forearm (events from both objects together), 34 trials for the outer part of the forearm, and 77 trials for the right hand. The results are shown in Fig 7 with the inner part of the left forearm on the left (627 samples, taxel nr. 2), the outer part in the center (451 samples; taxel nr. 8), and right hand on the right (944 samples; taxel nr. 2).

### Interim discussion on learning in the real robot

#### Comparison of representations learned in different scenarios

The experimental results detailed in the previous sections show comparatively similar outcomes for the representations learned on the same taxel (taxel nr. 2 of the internal part of the left forearm) subject to the different experimental conditions (tactile-motor, tactile-visual with double-touch and tactile-visual with external objects). However, there are some differences that are worth mentioning. Specifically, the representation learned in the tactile-motor scenario (Fig 4) shows a “crisper” landscape, which becomes progressively less defined in the subsequent sessions (Figs 5 and 7). This result is expected: as we demonstrated in Section Learning in a single taxel model, an increase of the noise in the input signal as well as in the variability of the stimulation results in a broader profile of the activation landscape (see Figs 2 vs. 3). The double-touch (tactile-motor) scenario is a highly controlled setup in which the robot performs a number of similar trials with similar velocity profiles, using an inverse kinematics solver and controller. By reducing reliance on the kinematics, and progressively depending on an intrinsically noisy sensory system (i.e. the visual system), the contribution of noise becomes more prominent. Further, training trials for the tactile-visual learning with external objects are performed by a human experimenter, with little control on the type of trajectories that are presented to the robot, resulting in a broader landscape of the probability function.

#### Comparison of representations learned by different body parts

**Fig 8:**
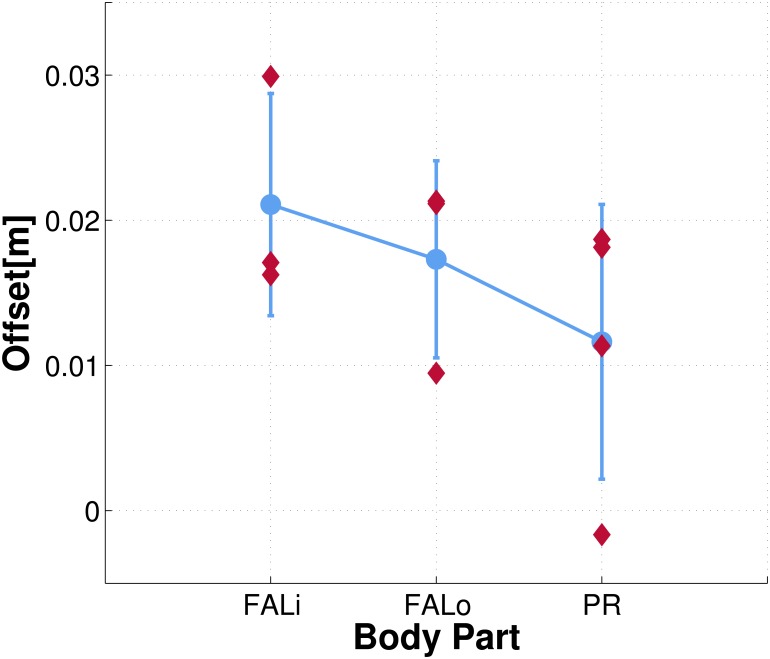
Systematic offsets computed during tactile-visual learning using external objects. The distance offset in the learned representation of ten taxels (three on the inner part of the left forearm, *FAL*_*i*_; three on the outer part, *FAL*_*o*_; four in the right palm, *PR*) is depicted in red. For each of the three body parts under consideration, average offset and standard deviation are depicted in blue.

In the Tactile-visual learning using external objects scenario, three different skin parts were subject to training: internal and external part of the left forearm, and the right hand (palm). Representations learned around selected taxels were shown in Fig 7. Here we look at aggregate statistics of all the taxels for each of the skin parts. We postulate that a significant component of the systematic error pertaining to a taxel is skin part specific and can be mainly attributed to the position on the kinematic chain (e.g. forearm vs. hand) and the mounting of individual skin patches (see Experimental Setup). In order to validate this hypothesis, we extrapolate the systematic offset of the ten virtual taxels that were stimulated during the experimental session and analyze the overall trend between different body parts. To this end, we performed a weighted orthogonal 2D least-squares regression, with [*x*, *y*] coordinates given by [*D*, *TTC*], and weights equal to the learned representation at each of the pairs (i.e. the contact probability, *f*(*D*_*i*_, *TTC*_*j*_), see Eq 3 under Internal representation). A weighted 2D regression applied to the 3D landscape reduces the dimensionality of the input space, and lets us evaluate at which distance *D* the regression line crosses the *x*−axis (i.e. *TTC* = 0)—giving the offset pertaining to the position of the particular taxel. Results are depicted in Fig 8: most of the taxels show an overall error between 1cm and 3cm, with an average error of 2.11cm and 1.73cm for the inner and outer part of the left forearm respectively, and 1.16cm for the right palm. The results suggest that the systematic errors depend on the specific skin part the taxels belongs to, even though additional “intra-skin-part” variance is present. Importantly, the learned representations automatically compensate for these errors as will be demonstrated later.

#### Analysis of the learning progress

**Fig 9:**
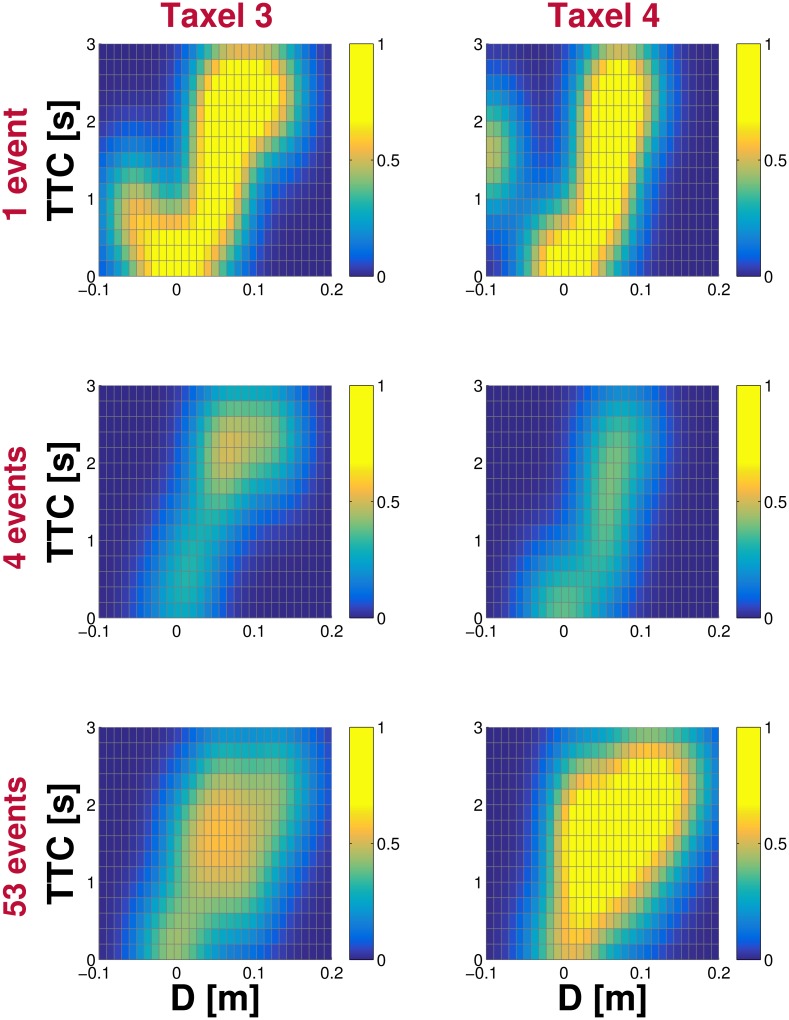
Evolution of the learning process. The progress of the learned representations belonging to two adjacent taxels of the internal part of the left forearm is shown. For each of the two taxels (taxel nr. 3 on the left column and taxel nr. 4 on the right column), snapshots of their respective representations after 1, 4 and 53 trials are depicted. See text for details.

As mentioned in Section Learning in the real robot, one of the features of the model is the ability of each taxel to learn a usable representation very quickly, from a few training samples. This is a direct consequence of the smoothing approach (Parzen windows applied to the discrete representation) for undersampled spaces. To illustrate this, in Fig 9 we show the evolution of the representations belonging to two neighboring taxels in the internal part of the left forearm during tactile-visual learning with external objects. Starting from a blank state for both taxels, we depict the representation after the same “positive” example (i.e. the nearing object contacted both taxels), after 4 examples (combination of positive and negative trials), and after the full training of 53 examples.

The results show how the same input trial (approaching stimulus) affects each taxel differently, because it gets projected on each taxel’s FoR in a slightly different manner. After the first trial, there is a clear bias toward the only experience the taxels had (the lack of negative examples practically translates into maximal certainty of collision prediction for some parts of the input space). Nonetheless, although the representations are far from being comparable to their respective final versions, even with a single (positive) example they can be already used for a coarse estimation of the probability of being touched by future incoming objects. Finally, after the full training session, the respective landscapes of the two adjacent taxels show similarities in both their shapes and offsets. Yet, taxel nr. 4 exhibits stronger responses over its landscape, which is a consequence of the fact that taxel nr. 3 is positioned closer to the robot’s wrist and it is thus less likely to be contacted and to experience positive trials. This illustrates the effect of the individual taxel’s training on the learned representation, which is further shaped by the embodiment—the taxel’s physical placement in this case.

### Exploitation of the learned associations

The learned representation is validated during an avoidance/reaching experimental session, corresponding to the last milestone: exploitation of learned associations (Table 4). The robot uses the acquired model in order to either avoid or come into contact with an incoming stimulus with any of the skin parts that have a peripersonal RF. Similarly to the learning stage, experiments are conducted by presenting the robot with a series of stimuli. An approaching object thus triggers the activation of each taxel given by the taxel’s previous “experience” with similar stimuli (in terms of [*D*, *TTC*]). Consequently, this gives rise to a distribution of activations pertaining to the skin surface. It is important to note the following: i) the iCub built up a PPS representation based on stimuli that are directed toward the skin; ii) in order to test these representations, we exploit a similar scenario, in which the robot has to either move away from or reach for approaching objects. Static objects (or objects that are moving *away* from the robot) do not trigger a response from the PPS representation and hence do not generate any movement, which is desirable and in accordance with neurophysiological data on approaching vs. receding stimuli (see e.g., Graziano and Cooke [2]).

The iCub is presented with an unknown object that was not used in the learning stage (a pink octopus). It is used by the experimenter to perform a series of approaching behaviors toward the robot’s body parts that had previously learned their representations (left forearm and right hand). The visual processing pipeline used was identical to the learning stage (see Section Visual processing and gaze control). However, here, the taxels’ activations are exploited by the robot to either avoid or “reach for” the approaching object with any of the body parts used during learning. Only taxels with activation above a certain threshold contributed to the resulting movement vector that was eventually executed by the controller. The threshold was empirically set to 0.4, corresponding to a 40% chance of that taxel being contacted by the nearing object (according to the learned model). In order to achieve the desired behavior, we implemented a velocity controller that can move any point of either the left or right kinematic chain of the arms in a desired direction. During an avoidance task, the movement is directed away from the point of maximum activation, along the normal to the local surface in that point. For “reaching”, the desired movement vector has the opposite direction. The setup of the controllers is described in Section Avoidance and reaching controller.

#### Margin of safety: Avoidance behavior demonstration

**Fig 10:**
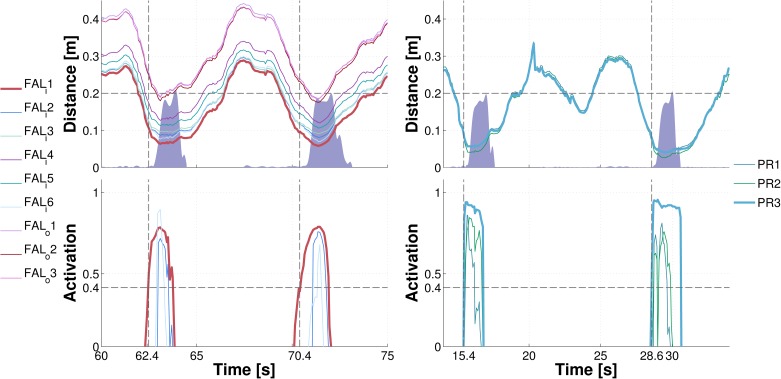
Avoidance demonstration. **(Left)** Object approaching the inner part of left forearm. Nine taxels of the left forearm (six on the inner part, *FAL*_*i*_ 1: 6; *FAL*_*i*_ stands for forearm left internal; three on the outer part, *FAL*_*o*_ 1: 3) were considered in the experiment. Top plot shows the distance of the object from the taxels in their individual FoRs. The shaded purple area marks the velocity of the body part (common to all taxels; maximum activation corresponding to 10*cm*/*s*). Bottom plot depicts the activations of the forearm taxels’ PPS representations. **(Right)** Object approaching the right palm. There were three taxels considered (*PR* 1: 3, where PR stands for “palm right”).

To demonstrate the performance of the avoidance behavior, we conducted an experimental session of roughly 20 min. in duration where we performed a series of approaching movements with a previously unseen object, the octopus toy, alternating between the body parts and varying the approaching direction. Avoidance behavior was successfully triggered in all cases. A snapshot illustrating typical behavior in a 15*s* window for the left forearm (Fig 10 left) and a 20*s* window for the right palm (Fig 10 right) is shown—with two approaching events in each plot. In total, nine taxels of the left forearm (six on the inner part; three on the outer part) and three taxels of the right palm were considered. The top plots show the distance of the approaching object from the individual taxels (in their respective FoRs). The bottom plots show the activations of the learned representations for each taxel (note that this representation uses a two-dimensional domain of [*D*, *TTC*]; however, to demonstrate the behavioral performance, we restrict ourselves to showing distance only in the upper plot). As the object comes closer, there is an onset of activation in the “most threatened” taxels. Once the activation level exceeds a predefined threshold (0.4 in this case—horizontal line in the bottom panels), the avoidance behavior is triggered. This is illustrated in the top plots with the shaded purple area that marks the velocity of the body part as a result of the avoidance controller command. The first taxel responding is highlighted in the corresponding upper and lower plots. The upper plots clearly demonstrate that the avoidance behavior was effective: a safety margin has always been preserved. Qualitatively similar behavior was observed during the whole experimental session. The controller takes advantage of the distributed representation pertaining to individual taxels, averaging the expected contact locus as well as its likelihood. This loosely resembles the way noisy information is averaged in neural population coding schemes (e.g., [50]).

#### “Reaching” with arbitrary body parts

**Fig 11:**
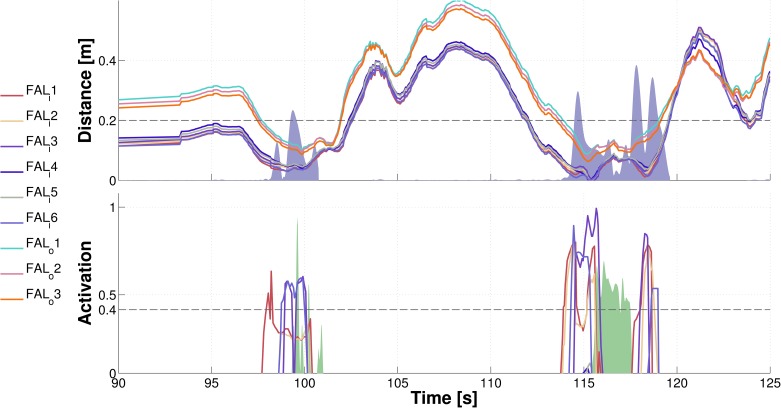
Reaching with arbitrary body parts demonstration. Object approaching the inner part of left forearm. Nine taxels of the left forearm (six on the inner part; three on the outer part) were considered in this experiment. Top plot shows the distance of the object from the taxels in their FoRs. The shaded purple area marks the velocity of the body part due to the activation of the controller. The bottom plot depicts the activations of the forearm taxels’ representations. The green shaded area marks physical contact with the robot’s skin—aggregated activation of all tactile sensors contacted on the body part.

In a similar fashion, we tested the “reaching” controller in a session of approximately 10 min. in duration. Note that this is “reaching” not in a traditional sense of reaching with the end-effector—the hand. Instead, the particular skin area most likely to collide with the stimulus will be recruited to “reach” or “catch” it. A snapshot illustrating the performance while approaching the inner part of left forearm is shown in Fig 11. The graphical illustration is the same as in the avoidance case. The spatial representations pertaining to the taxels get activated (bottom plot) and trigger the movement, which in this case is approaching the object. In addition, the bottom plot illustrates the physical skin activation (green shaded area). Importantly, contact is generated in both cases as the skin activation testifies. The fact that the distance is greater than zero in the first event can be attributed either to errors in the visual perception or to an offset in the kinematic transformations.

#### Joint space and operational space range

During tactile-motor and tactile-visual training using “double-touch”, the robot controls both arms to satisfy the self-touch constraint, thus automatically attaining different—even if somewhat stereotypical—arm configurations. Conversely, the tactile-visual learning using external objects was performed in a static configuration—the robot is passively waiting for external objects to contact the skin. Nonetheless, exactly the same software is used in all cases, since it automatically handles any configuration. The robustness of our approach to different arm configurations is even more evident in the subsequent exploitation of the learned associations, which involved the richest repertoire of configurations. The movement response is always different, depending on where the object is coming from and which portion of the peripersonal space representation is activated the most. To illustrate the range of different configurations, we have extracted in Table 6 the extremes reached by individual degrees of freedom (DoFs). It is evident how most of the joints actively involved in the movements (the shoulder joints and the elbow) have spanned a large portion of their range, with some of them even covering all of their operational range. In addition, we quantified the range of the end-effectors in the operational space (see Table 7, with further details provided in S1 Fig). For safety reasons, the range of the end-effectors during the experiment was artificially restricted to be confined to a sphere of radius 0.2 *m* around the home position. Also, note that the data was recorded only while the peripersonal space representation was active—i.e. while activations were exceeding a threshold. In summary, this demonstrates that the representations learned were robustly activated in a wide range of joint configurations and end-effector positions.

**Table 6:** Range of arm DoFs during avoidance and reaching. Each of the 7 DoFs that belong to the left and right arms are depicted: 3 DoFs for the shoulder (s_0_, s_1_, s_2_), one elbow joint (e_0_) and three joints pertaining to the wrist (w_0_, w_1_, w_2_). For each joint, its minimum and maximum angles are shown, along with its range. Joints s_2_ and e_0_ of the left arm, as well as joint s_2_ of the right arm, reached their full physical limits during the experiments. Wrist joints did not contribute to either the avoidance or the reaching behaviors.

**Left Arm [deg]**							
	s_0_	s_1_	s_2_	e_0_	w_0_	w_1_	w_2_
Min	-65.0	19.5	-39.2	16.3	-0.88	-0.015	-0.025
Max	7.1	64.2	80.1	106.0	1.38	0.064	0.063
Range	72.1	44.7	119.9	89.7	2.26	0.079	0.088
**Right Arm [deg]**							
	s_0_	s_1_	s_2_	e_0_	w_0_	w_1_	w_2_
Min	-61.7	18.6	-37.8	15.5	-1.57	-0.079	-0.085
Max	7.9	45.5	80.7	80.5	0.86	0.111	0.159
Range	69.5	26.8	118.5	65.0	2.43	0.190	0.244

**Table 7:** End-effector extremes in operational space during avoidance and reaching. For both the left and right end-effectors, the minimum and maximum values reached in the *x*−, *y*− and *z*− axis are shown, along with its range of operation. For safety reasons, the operational space of the robot was constrained within a sphere centered in the resting position (set to [−0.30, −0.20, +0.05] *m* for the left arm and [−0.30, +0.20, +0.05] *m* for the right arm—in iCub Root FoR) and with radius equal to 0.2 *m*. Please refer to S1 Fig for a depiction of the robot’s kinematics during the avoidance and reaching scenario.

Left End-Effector [m]				Right End-Effector [m]			
	*x*	*y*	*z*		*x*	*y*	*z*
Min	-0.34	-0.39	-0.06	Min	-0.34	0.06	-0.03
Max	-0.18	0.00	0.15	Max	-0.19	0.36	0.10
Range	0.15	0.40	0.22	Range	0.15	0.29	0.13

#### Comparison with model without TTC information

In this section, we compare the proposed approach with our previous work [45]. In particular, in this work we benefit from a richer representation because of the introduction of the time-to-contact (TTC) dimension. Although this results in a more complex model and the need to increase the number of training samples in order to converge to a stable representation, we believe that the information carried out by the TTC is crucial in the construction of a model of nearby space that is meaningful and effective in a real world scenario. Specifically, by including dynamic information about the speed of the approaching object, the proposed model can easily distinguish which objects pose an immediate threat to the body. To make a practical example, the TTC of a close but static object would be infinite, whereas it would be negative for an object that is moving away from the skin; in both cases, such objects would be easily discarded because they would not fall within the boundaries of our representation, which considers objects with a TTC included in the range [0; 3*s*]. The exploitation of this feature can be demonstrated by comparing the avoidance and “reaching” controllers in this work and [45]. Without loss of generality, in the following we compare only the avoidance behaviors, although similar conclusions can be drawn by analyzing the “reaching” with arbitrary body parts controllers. Fig 10 shows how the taxel of interest is activated only when the object is approaching it, i.e. when its distance decreases over time. When the object is moved away by the experimenter (approximately at *t* = 64*s* and *t* = 71*s* in Fig 10 left), the taxels become silent and the avoidance behavior stops. A comparison with previous work—see S2 Fig—, instead, shows how this is not the case if only the distance is taken into account: the taxels’ activation fades completely only if the object moves away enough to fall out of the receptive field, i.e. farther than 20*cm* from the skin.

## Discussion and Conclusion

In this paper, to the best of our knowledge, we presented the first robot that learns a distributed representation of the space around its body by exploiting a whole-body artificial skin and either self or environment physical contact. More specifically, each tactile element has been associated to a spatial receptive field extending in the 3D space around the skin surface. Stimuli in the form of motor or visual events are detected and recorded. If they eventually result in physical contact with the skin, the taxels update their representation tracing back in time the approaching stimulus and increasing the quality of the internal probability estimate—in terms of distance and time to contact—that is, an estimation of the likelihood that the stimulus eventually touches any given body part. The spatial RF around each taxel is constructed and updated as the limbs move in space by combining the joint angles and knowledge of the robot’s kinematics; however, its representation is adapted from experience, thus automatically compensating for errors in the model as well as incorporating the statistical properties of the approaching stimuli. This representation naturally serves the purpose of predicting contacts with any part of the body of the robot, which is of clear behavioral relevance. Furthermore, we implemented an avoidance controller whose activation is triggered by this representation, thus endowing the iCub with a “margin of safety”. Finally, simply reversing the sign of the controller results in a “reaching” behavior toward approaching objects, using the closest body part.

One important feature of the proposed method is its invariance with respect to the robot configuration (posture) and the input modality used. Capitalizing on the robot’s kinematic model, current stimulus positions are automatically remapped into every taxel’s FoR, taking also every taxel’s current pose (position and orientation) into account. In the double-touch scenario, both the “receiving” arm with the taxel array and the “touching” arm with the extended finger (the nearing stimulus) move, which, however, does not pose any difficulty for our method. Furthermore, our model is agnostic as to whether the stimulus was perceived motorically or visually. In the last scenario with external approaching objects, the arm configuration was static during learning, but the head and eyes were moving. Nevertheless, a moving arm would again be automatically considered using exactly the same computation. This is also demonstrated in the avoidance / “reaching” scenarios, where the arm moves, but the stimulus’ effect on the taxels is constantly evaluated, resulting in online adaptation of the robot response.

Another important asset of the proposed model is that learning is fast, proceeds in parallel for the whole body, and is incremental. That is, minutes of experience with objects moving toward a body part produce a reasonable representation in the corresponding taxels that is manifested in the predictive prior to contact activations as well as in the avoidance behavior. Smoothing using Parzen windows applied to the discrete representation specifically contributes to this effect in the case of undersampled input spaces.

The investigated scenarios parallel those experienced by humans and animals—also because of the anthropomimetic nature of the iCub—and should thus inform us directly about the mechanisms of peripersonal space representations in primates as they have been subject of intensive investigations in cognitive psychology as well as the neurosciences over decades. First, the developmental trajectory leading to the acquisition of these representations is largely unknown. The development of reaching (e.g., [51, 52]) may constitute one key factor in this mechanism; the exploration of own body may be another (e.g., [11, 17]). In this paper, we mimicked a similar developmental trajectory by considering first the self-touch behaviors and adding encounters with objects later on.

The architecture presented is, at this stage, not a model of a particular brain network. Casting it into the vocabulary common in the neurosciences, one could say that the representation associated with every taxel may correspond to a spatial receptive field of a neuron that is centered on that particular taxel (hence body-part centered coordinates). The RF has a fundamentally spatial nature; further, it is modality-independent—as we demonstrated by entering it and eliciting its “neural” response with motor/proprioceptive as well as visual targets. However, note that this “neuron” does not have a tactile RF—tactile sensations were used in the learning/adaptation of this RF only. However, it would be easy to extend our representation by constructing a bimodal visuo-tactile or, more precisely, tactile-spatial neuron whose activation would be the sum of the “spatial” and tactile inputs. The reference frame transformations are in our case mediated by the kinematic model of the robot and use the iCub Root FoR as common ground connecting all kinematic and visual chains. This is unlikely to correspond to the exact mechanism used by the brain; however, bimodal neurons with tactile RFs on a body part and visually RFs around it and anchored to it—following it in space independently of eye position—have been identified both in premotor areas (F4) (e.g., [53] for a survey) and parietal areas (VIP and other—e.g., [23]). Our position is similar to [29], for example, assuming that the necessary coordinate transformations (from visual or proprioceptive input to body-part centered coordinates) are performed by an upstream process. Our model then receives this as input. Several common reference frames (e.g., eye-centered [54]) have been proposed to act in the posterior parietal cortex. In summary, the architecture presented is a first implementation that supports the relevant behaviors. However, since the scenarios as well as the sensory modalities available to the robot parallel the conditions in biology (at a certain level of abstraction), the road is open to further grounding of the architecture to the corresponding putative brain mechanisms.

One possible practical limitation of the presented architecture is its computational and memory requirements. The distributed and parallel nature of the representation has many advantages. At the same time, the complexity grows linearly with the number of taxels—each of them monitoring its spatial receptive field and, possibly, updating its probabilistic representation. However, this is clearly in line with the nature of brain computation. Furthermore, the spatial resolution we have selected (with taxels of around 2*cm* in diameter on the skin surface) is likely unnecessarily high—the body-part-centered receptive fields of parietal cortex neurons are typically much larger (e.g. spanning a whole upper arm [5]). Also, lower resolution may still suffice to support the margin of safety behavior. Such a modification would be straightforward in our setup, requiring only a redefinition of the virtual taxels.

The “demonstrators”—avoidance and “reaching”—are also only first steps in this direction. They are simply exploiting fairly standard controllers to generate movements of a virtual point that is a result of voting of taxels activated by a moving object. Avoidance differs from reaching in the direction of this movement vector only. This could be further differentiated and developed, leading to simple reflexive as well as complex whole-body avoidance mechanisms such as those reported in monkeys [2]; an implementation in the iCub relying on force/torque feedback has been presented in [41]. Finally, “reaching” here is a simple mechanism that results in approaching to a nearing object with the skin part that was most likely to be contacted by the object. Yet, this resembles the principle of motor equivalence, where the controller in fact can generate reaching movements using arbitrary body parts as end-point.

Future work can proceed along several directions. First, the architecture can be refined and better grounded in concrete mechanisms that are assumed to operate in the primate brain, leading to a better explanation of why certain connectivity patterns including polymodal neurons are a necessity and not only the result of the quirks of evolution. This would provide an invaluable tool to test biological theories and crucially advance the computational modeling efforts. Second, the full kinematic model of the robot that was taken for granted here could be dropped and the learning problem expanded to full complexity dealing with the emergence of spatial representations from motor, proprioceptive, tactile and visual inputs. The double-touch scenario could in fact serve this very purpose of body schema learning; the self-calibration framework of [18] could be adapted and the Denavit-Hartenberg representation of kinematics and the inverse kinematics solver replaced by more biologically motivated analogs. Third, the margin of safety in primates does not have uniform extension and resolution; instead, body parts, in particular face and hands, receive more attention than others. This could be emulated in the robot as well. Fourth, the model proposed in this work could be further developed to address the expansion of the RFs after tool use as first documented by [34] and modeled by [33] in a humanoid robot. Fifth, the architecture proposed is prone to impact on practical applications. Whole-body tactile sensing together with a virtual margin of safety around the robot’s body dramatically increases the robot’s own safety as well as safety of humans that share the environment with the robot. The proposed implementation will have to be tested in such scenarios and possibly enhanced also by force/torque sensing that is already available on the iCub to guarantee robustness in all situations. Finally, with the advent of robotic skin technologies (see [39] for a review), frameworks similar to this one can find applications in diverse robotic platforms and are by no means restricted to the iCub humanoid robot (or to humanoids altogether).

## Materials and Methods

### Experimental Setup

The iCub is a full humanoid robot platform originally developed to support research in artificial cognitive systems. In this section we describe the key components relevant for this work: the artificial skin, the robot’s sensing modalities, the eye and camera setup, the model of the robot’s kinematics, visual processing and gaze control, and finally the avoidance and reaching controller used in the experiments. For details on the basic structure of the iCub we refer the reader to [55].

#### Artificial skin

**Fig 12:**
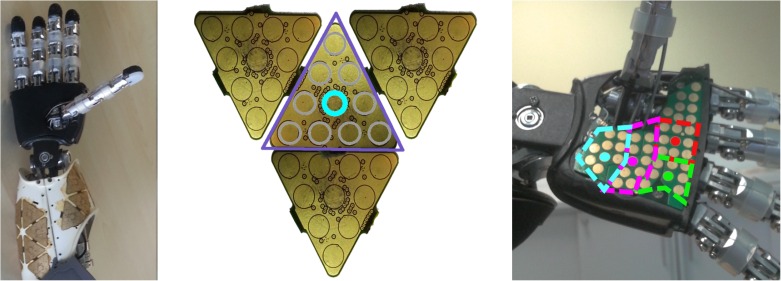
Pressure-sensitive skin of the iCub. **(left)** iCub forearm with exposed skin patches. **(middle)** Four triangular modules of the iCub skin PCB (10 taxels each). The central taxel corresponds to the virtual taxel which in turn is made by joining the responses of a full triangle. **(right)** Exposed skin of the palm with virtual taxels highlighted.

Recently the iCub has been equipped with an artificial pressure-sensitive skin covering most body parts [26]. The latest iCub version contains approximately 4000 tactile elements (taxels)—in the fingers, palms, forearms and upper arms, torso, legs and feet. In the experiments performed in this work, we restrict ourselves to the forearms and palms. The iCub forearm and hand with exposed skin is shown in Fig 12 left. With the exception of the palm and fingertips, the skin covering all body parts consists of patches with triangular modules of 10 taxels each (Fig 12 middle). There are in total 23 modules on the forearm in two patches and hence 230 taxels. However, for the purposes of this study this resolution would be an unnecessary burden. Therefore, we generate RFs grouping all responses in a triangular module in a single “virtual” taxel whose position in the body surface corresponds to the central physical taxel.

The palm has a slightly different structure corresponding approximately to four triangles (see Fig 12 right). It is made out of a single printed circuit board composed of an array of 43 taxels. We artificially split the array into four regions of 8 to 10 taxels, forming virtual taxels as before. These are shown in Fig 12 (right), with the central taxels marked with full circles. The region enclosed between the thumb and the fingers is not considered as it is unlikely that it is touched by a moving object. In the main article, we use “taxel” to refer to this virtual taxel.

A spatial calibration of the skin of the forearm with respect to the iCub kinematic model has been performed in del Prete et al. [48]. The palm was calibrated using data from the CAD model. The position of all taxels as well as the orientation of their surface normal in the iCub Root FoR can thus be extracted if the current joint configuration is known.

#### Joint angle sensing

Proprioceptive inputs in the iCub simply consist in angular position measurements at every joint. For most joints, they are provided by absolute 12bit angular encoders (see [55] for details); small motors (head and hands) employ incremental encoders whose zeros are calibrated at startup.

#### Head and eyes

Vision of the iCub is provided by two cameras mounted in the robot’s eyes. The neck of the robot has three degrees of freedom (DoFs) and there are three additional DoFs in the eyes allowing vergence and version behaviors. The tilt DoF is mechanically coupled (both eyes move up and down); the version and vergence movements are coupled in software following an anthropomimetic arrangement. With appropriate calibration [56], depth information can be extracted from binocular disparity.

#### Kinematic model and coordinate transformations

The iCub sensors provide raw data in different FoRs. These need to be transformed in order to compare similar quantities. In primates, the role of establishing a common ground between these rich but diverse sources of information is attributed to the body and peripersonal space representations. As we described in Section Introduction, coordinate transformations (such as between eye-centered and body-part-centered FoRs) are necessary. In our case, we specifically need two types of transformations:

- *Purely kinematic transformations*. For the first scenario where the robot learns about the space around its body through double-touch (cf. Table 1), the “touching” body part (like the index finger of the right hand—Fig 1 middle) need to be brought to the FoR of the taxels that are “touched” (like the skin on the left forearm).
- *Visual-kinematic transformations*. In the second scenario, vision is considered. There are two variants of the experiment: i) double-touch with visual tracking of the finger approaching the contralateral arm (see Table 2); ii) an independently moving object approaching and touching the robot’s skin (cf. Table 3). In both cases, transformations from the image (retina) FoR are necessary. This involves exploiting binocular disparity to obtain a 3D position of the object (or finger) in the head FoR and then following a sequence of coordinate transformations to eventually reach the FoR of individual taxels.

Learning these transformations was not the goal of this work; therefore, we have employed the existing kinematic model of the iCub that is based on the Denavit-Hartenberg convention and available in the form of a software library (*iKin*, [57]). The library allows traversing any kinematic chain of the iCub by employing an appropriate sequence of transformations. In fact, kinematic representations of individual chains in iKin start/end in the Root FoR of the robot (around the robot’s waist) and this is employed as an intermediary to connect individual sub-chains. The transformation to individual taxels’ FoRs is provided by the skin calibration.

These composite transformations are subject to errors that include (i) mismatch between the robot model based on the mechanical design specifications (CAD model) and the actual physical robot; (ii) inaccuracies in joint sensor calibration and measurements; (iii) unobserved variables as for example backlash or mechanical elasticity; (iv) inaccuracies in taxel pose calibration; (v) errors in visual perception due to inaccurate camera calibration. The combination of these sources of error can amount to a total of several centimeters. However, in the proposed approach, the representation that every taxel learns with regard to its surrounding environment will automatically compensate for the systematic component of these modeling errors.

#### Visual processing and gaze control

**Fig 13:**
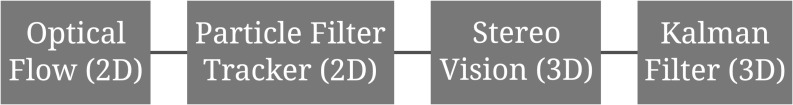
Tracking of generic objects. See text for details.

For the scenarios involving tactile-visual learning (described in Tables 2 and 3), additional processing steps are needed to compute the approaching stimuli’s position and velocity: moving objects need to be detected, segmented out of the background and their position tracked. We implemented two variants of the tracking mechanism:

1. **Tracking of fingertip with colored marker**. In this case, we implemented an HSV-based color segmentation module that can track a green marker placed on the iCub’s index fingertip on both the right and left image. A simple triangulation procedure yields the 3D coordinates of the fingertip in the robot’s Root FoR.
2. **Tracking of generic objects**. In this second case, we used a tracker for generic objects under certain moderate assumptions on the availability of visual features and limits on their velocity and size, as developed in Roncone et al. [45]. The tracking software consists of a number of interconnected modules, schematically depicted in Fig 13. The first module uses a 2D Optical Flow [58] to detect motion in the image. If this is the case, it triggers a 2D particle filter module [59] to track the object in the image plane based on its color distribution. At this stage, the 2D planar information related to the approaching object (namely, the centroid of the object and an estimation of its size) is converted into 3D (world) coordinates via a stereo disparity module [60]. A Kalman filter then completes the position estimation process. It uses 3D points as determined by the stereo vision module and it employs a fourth order dynamical model of the object motion. Finally, a gaze controller was employed in order for the eyes and head to smoothly follow the tracked object in space. The details of the gaze controller can be found in [20].

### Data collection for learning

**Fig 14:**
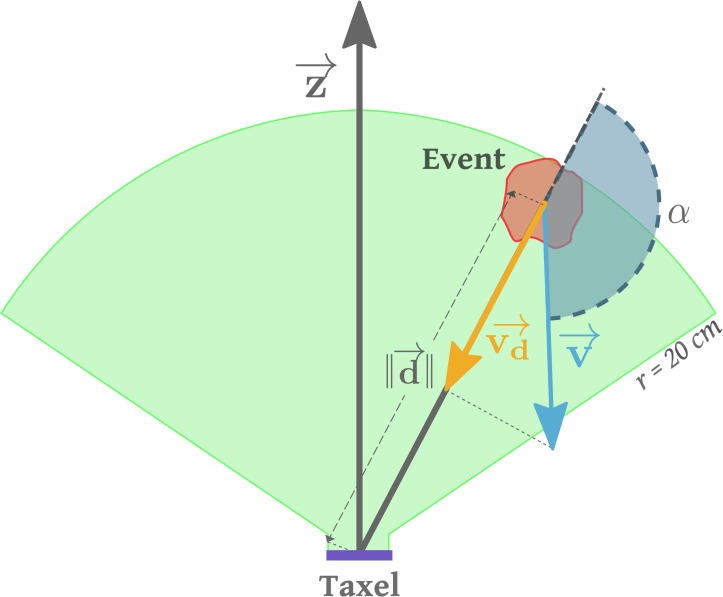
Receptive field of a taxel and approaching stimulus. See text for details.

As outlined above, two distinct scenarios were considered where a given skin patch was stimulated by the robot’s own body (double-touch) or by independently moving objects. However, the basic principle is the same, that is, in both cases it is implemented in a local, distributed, event-driven manner. An illustration of the two cases is depicted in Fig 1 (middle and right). A volume was chosen to demarcate a spatial “receptive field” (RF) around each taxel (we will use this notion of receptive field for the scenario in the robot from now on). Similarly to what happens in humans and monkeys, these receptive fields distributed around the body are anchored to the body part they belong to and encode local information. However, unlike in biology where receptive fields of individual neurons are tied to a particular sensory modality and response properties of the neuron, our receptive field is a theoretical construct—a volume of space around the taxel. In what follows, any stimulus moving toward the robot’s body—note that this can be either another part of the body of the robot or an external visual stimulus—will be remapped into the taxel’s reference frame and thus potentially enter its receptive field. The RFs are limited to a conical volume oriented along the normal to the local skin surface and extend to a maximum of 20cm from the surface (green region in Fig 14). This is consistent with neurophysiological observations [2]. When a stimulus enters the conical volume of a RF, we mark the onset of a potentially interesting event. Subsequently, the position and velocity of the object w.r.t the taxel is recorded and the distance *D* and time to contact *TTC* computed. The scalar distance, *D*, is calculated as follows:

$$\begin{array}{c} D=\mathrm{sgn}\left(\vec{d}\cdot \vec{z}\right)\left|\right|\vec{d}\left|\right|\;, \end{array}$$ (1)

where $\vec{d}$ is the displacement vector pointing from the taxel to the stimulus (geometric center of the incoming object), $\vec{z}$ is the *z-*axis of the reference frame centered on the taxel and pointing outward (coincident with the normal to the skin surface at the taxel position). The sign of the dot product is positive if the object lies in the hemisphere extending from the taxel. The scalar distance, *D*, preserves information about the relationship of the event w.r.t. taxel normal. *D* can be negative because of modeling or measurement errors or simply because a stimulus is behind a particular body segment. The time to contact, *TTC*, is defined as follows:

$$\begin{array}{c} TTC=-\mathrm{sgn}\left(\vec{d}\cdot \vec{v}\right)\frac{\left|\right|\vec{d}\left|\right|}{\left|\right|\vec{v_{d}}\left|\right|}=-\mathrm{sgn}\left(\vec{d}\cdot \vec{v}\right)\frac{\left|\right|\vec{d}\left|\right|}{\left|\right|\vec{v}\cdot \cos\left(\alpha \right)\left|\right|}\;, \end{array}$$ (2)

where $\vec{d}$ is again the displacement vector pointing from the taxel to the stimulus, $\vec{v_{d}}$ is the projection of the stimulus’s velocity $\vec{v}$ onto $\vec{d}$, and *α* is the angle between $\vec{v}$ and $\vec{d}$, as shown in Fig 14. The sign term takes into account the direction of motion of the stimulus. That is, for stimuli in the “positive hemisphere” moving toward the taxel, the dot product will be negative ($\vec{d}$ and $\vec{v}$ have opposite directions) and the time to contact will be positive. The opposite holds for objects moving away from the taxel or the case when modeling errors return a negative distance. The magnitude of *TTC* is simply distance over speed (norms of the respective vectors, $\vec{d}$ and $\vec{v_{d}}$).

This definition of *D* and *TTC* is clearly an approximation that simplifies the estimation of probability densities by bringing down the full description of a stimulus motion into a bi-dimensional space. This is useful to keep the learning procedure feasible with a small number of data points and it has the additional advantage of allowing one-shot learning: a single stimulus and contact with the skin enables a rough but useful estimation of the corresponding RF density. Note that this procedure—the recording of *D* and *TTC* of approaching stimuli—is carried out in parallel for all taxels whose RFs overlap with the stimulus location. These data are buffered for three seconds and used for learning only if the stimulus eventually touches the skin and at least one taxel measures the contact event. In this case, a learning iteration is triggered as follows:

1. For all the taxels that measured a contact event, the buffer of object positions in their local FoR is traversed back in time in time steps of 50 ms. As long as the stimulus is in a given RF, *D* and *TTC* at every time step are recorded as positive examples on each taxel’s data set.
2. For all other taxels of the same body part, the procedure is analogous, but negative examples are appended to their respective data sets.

Stimuli that move close to but never touch the body surface do not contribute to the peripersonal space representation. However, taking into account all events that come sufficiently close to the body surface would be an equally valid approach.

### Internal representation

Each taxel stores and continuously updates a record of the count of positive and negative examples that it has encountered for every combination of distance and time to contact. We defined the range of *D* as [−10, 20] cm and *TTC* as [0, 3] s. The variables were discretized into eight equally-sized bins for the distance and four bins for the time to contact respectively; the *TTC* requires a velocity estimation of the approaching object and gives rise to noisier estimates. There are 32 combinations and hence 32 items, [*n*_*positive*_, *n*_*negative*_], in every taxel’s memory. As mentioned earlier, the main advantage of this representation is its simplicity and the possibility of incremental updates—for each new positive or negative example, the respective count in memory is incremented. However, most relevant for the robot is an estimation of the probability (density) of an object hitting a particular part of the skin, which can be used to trigger avoidance responses, for example. The stimulus’s “coordinates” w.r.t. each taxel (i.e. distance, time to contact) can be discretized as described above and a frequentist probability estimate obtained simply as:

$$\begin{array}{c} P\left(D,TTC\right)\approx f\left(D,TTC\right)=\frac{n_{positive}\left(D,TTC\right)}{n_{positive}\left(D,TTC\right)+n_{negative}\left(D,TTC\right)} \end{array}$$ (3)

Such an approach—discretized representation and querying—constitutes the simplest solution. However, it may give rise to unstable performance, in particular in the case when the state space is undersampled. Therefore, it is desirable to obtain a continuous function *f* which can be sampled at any real values of [*D*, *TTC*]. This can be achieved by using the Parzen-Window density estimation algorithm [61]—in fact, to interpolate the data. In a 1-dimensional case, the interpolated value *p*(**x**) for any **x** is given by:

$$\begin{array}{c} p\left(x\right)=\frac{1}{n}\sum_{i=1}^{n}\frac{1}{h^{2}}\Phi \left(\frac{x_{i}-x}{h}\right) \end{array}$$ (4)

where **x**_*i*_ are the data points in the discrete input space, Φ is the window function or kernel and *h* its bandwidth parameter, which is responsible for weighting the contributions of the neighbors of the point **x**. We used a Gaussian function, hence we have:

$$\begin{array}{c} p\left(x\right)=\frac{1}{n}\sum_{i=1}^{n}\frac{1}{\sqrt{2\pi }\sigma }\exp\left(-\frac{{\left(x_{i}-x\right)}^{2}}{2\sigma^{2}}\right) \end{array}$$ (5)

In our case, which is bi-dimensional (with **x** = [*D*, *TTC*] as the input variables), we specified the standard deviation *σ* equal to the width of the single bin in each dimension of the input space. For any value of *D* = *d* and *TTC* = *ttc*, the final interpolated value, *p*(*d*, *ttc*), represents the probability of an object at distance *d* and time to contact *ttc* hitting the specific taxel under consideration. It is worth noting that only the original discretized [*D*, *TTC*] combinations have estimates of a probability function associated with them, each pair [*D*_*i*_, *TTC*_*j*_] independently from others. However, the whole “landscape” arising from *f*(*D*, *TTC*) cannot be interpreted as a probability mass function (in discrete case) or probability density function (in continuous case), because the overall probability for the whole space of *D* and *TTC* combinations can take any values and does not sum to 1.

### Monte Carlo simulation of a single taxel

**Fig 15:**
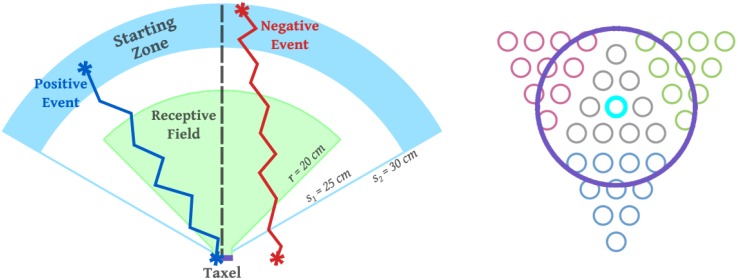
2D schematics of single taxel model. **(Left)** Side view of the simulated taxel with examples of approaching stimuli: the purple slab at the bottom represents a taxel; the green sector is a projection of the taxel’s cone-shaped receptive field; blue sector marks the region where stimuli are generated. The two examples show a positive event (blue line) and a negative one (red line). **(Right)** Top view of the simulated taxel with its nearby skin structure: the purple circle represents grouping of several sensors (physical taxels) in a modeled taxel (virtual).

In order to investigate the quality of the representation proposed in Section Internal representation, a Monte Carlo simulation was implemented. In particular, we wanted to study the properties of the acquired representation in noiseless and noisy conditions—with sufficient samples available and with control over noise—and investigate the effect of the hyper-parameters (such as number of bins for discretization, definition of the RF cone, range of stimuli’s speed, etc.). To this end, a 3D model of a single taxel with simulated stimuli was prepared—see Fig 15 for an illustration of the simulation environment. The code with the complete model is available at the public repository [47].

The model parameters were chosen to mimic the real robot setup as closely as possible. The simulated taxel itself has a radius of 0.235*cm*, which mimics the radius of the real iCub taxels. However, objects landing within 2*cm* from the taxel’s center (purple areas in Fig 15) are still considered positive, resembling the size of a triangular module of the iCub skin which is itself composed of 10 taxels (see Fig 12 above). These “virtual taxels” will be used in the real setup by joining the responses of a number of adjacent physical taxels. The taxel’s cone-shaped receptive field is depicted in green. Approaching stimuli are simulated by generating trajectories possibly corrupted by measurement noise. Since the nature of our data collection and learning method requires positive examples (objects contacting the virtual taxel) as well as negative examples (objects contacting neighboring taxels), we simulated three neighboring virtual taxels (Fig 15 right). We implemented a stochastic “shower” of objects with their starting points uniformly distributed in the blue region (“starting zone” in Fig 15 left) and their landing points following a Gaussian distribution centered on the simulated taxel (*μ* = 0; *σ* = 5*cm*). The velocity of the object is a vector directed from the starting point to the landing point, whose speed is uniformly distributed between 5*cm*/*s* and 15*cm*/*s* (but constant over time for any given trajectory). Position and velocity are sampled with *T* = 50*ms*. Measurement noise is Gaussian both for position and velocity. The Monte Carlo simulation is implemented in Matlab.

### Avoidance and reaching controller

As an exploitation of the learned representations, we implemented a velocity controller that can move any point of either the left or right kinematic chain of the arms in a desired direction. During an avoidance task, the movement is directed away from the point of maximum activation, along the normal to the local surface in that point. For reaching, the desired movement vector has the opposite direction. We compute a weighted average for both the position of the avoidance/reaching behavior and its direction of motion as follows:

$$\begin{array}{c} \begin{array}{cc} P(t) & =\frac{1}{k}\sum_{i=1}^{k}\left[a_{i}\left(t\right)\cdot p_{i}\left(t\right)\right]\\ N(t) & =\frac{1}{k}\sum_{i=1}^{k}\left[a_{i}\left(t\right)\cdot n_{i}\left(t\right)\right] \end{array} \end{array}$$ (6)

where **P**(*t*) and **N**(*t*) are the desired position and direction of motion in the robot’s Root FoR respectively, **p**_*i*_(*t*) and **n**_*i*_(*t*) are the individual taxels’ positions and normals. These are weighted by the activations, *a*_*i*_(*t*), of the corresponding taxels’ representations. The weighted average is computed by cycling through all the taxels whose activation is bigger than a predefined threshold at any given instant of time. Therefore, the resultant position and the direction of motion of the avoidance/reaching behavior are proportional to the activation of the taxels’ representations and change dynamically as the activation levels of different taxels varies. The velocity control loop employs a Cartesian controller [57] whose reference speed was fixed to 10*cm*/*s*.

## Supporting Information

S1 Fig End-effector trajectories in operational space during avoidance (red) and reaching (blue). A schematic illustration of the robot’s upper body kinematics during resting configuration is depicted. For each link—torso (gray), left arm (pink), right arm (light blue), right and left eye (yellow)—the end-effectors’ reference frames are also shown.
(EPS)

S2 Fig Avoidance demonstration using distance only information. **(Left)** Object approaching the inner part of left forearm. Top plot shows the distance of the object from the taxels in their individual FoRs. The shaded purple area marks the velocity of the body part (common to all taxels; maximum activation corresponding to 10*cm*/*s*). Bottom plot depicts the activations of the forearm taxels’ PPS representations. First approaching behavior was directed to the external part of the forearm (taxels in tones of green); second approach toward the internal part (taxels in tones of red) **(Right)** Object approaching the right palm. From [45].
(EPS)
